# Salt Content Impacts Food Preferences and Intake among Children

**DOI:** 10.1371/journal.pone.0053971

**Published:** 2013-01-16

**Authors:** Sofia Bouhlal, Claire Chabanet, Sylvie Issanchou, Sophie Nicklaus

**Affiliations:** 1 CNRS, UMR6265, Centre des Sciences du Goût et de l'Alimentation, Dijon, France; 2 INRA, UMR1324, Centre des Sciences du Goût et de l'Alimentation, Dijon, France; 3 Université de Bourgogne, Centre des Sciences du Goût et de l'Alimentation, Dijon, France; Université Lyon, France

## Abstract

Decreasing dietary sodium intake, which can be achieved by reducing salt content in food, is recommended. Salt contributes to the taste of foods and makes them more enjoyable. Whether a food is liked or disliked is an important determinant of food intake, especially among children. However, the role of salt in children's food acceptance has received little attention. The impact of salt content on children's hedonic rating and intake of two foods was investigated in children. Using a within-subject crossover design, we recruited 75 children (8–11 years) to participate in five lunches in their school cafeteria. The target foods were green beans and pasta. The added salt content was 0, 0.6 or 1.2 g/100 g. The children's intake (g) of all lunch items was measured. The children provided their hedonic rating of the food, a preference ranking and a saltiness ranking in the laboratory. Children could rank the foods according to salt content, and they preferred the two saltier options. A food-specific effect of salt content on intake was observed. Compared to the intermediate level (0.6 g salt/100 g), not adding salt decreased green bean intake (−21%; p = 0.002), and increasing the salt content increased pasta intake (+24%; p<0.0001). Structural Equation Modeling was used to model the relative weights of the determinants of intake. It showed that the primary driver of food intake was the child's hunger; the second most important factor was the child's hedonic rating of the food, regardless of its salt content, and the last factor was the child's preference for the particular salt content of the food. In conclusion, salt content has a positive and food-specific effect on intake; it impacted food preferences and intake differently in children. Taking into account children's preferences for salt instead of their intake may lead to excessive added salt.

## Introduction

The relationship between dietary sodium (Na) intake and the prevalence of some diet-related diseases has long been recognized [1], [2]. Thus, reducing dietary sodium intake has become a public health priority in several countries. Consequently, some governments have enacted policies aimed at reducing the salt (NaCl) content of foods [3], as sodium levels are often increased by salt in food (1 g of NaCl = 400 mg of Na). Given the importance of childhood in establishing food preferences and healthy eating habits [4], [5], analyzing the role of salt in children’s food preferences and intake is important. The recommended salt intake for children aged 8–11 years is 5 g per day [6]. In France, salt intake in this population is 6 g per day and increases with age [7]. Salt contributes to the taste of foods and makes them more enjoyable [8]. For children, the role of food sensory properties (i.e., taste, texture, smell) on eating behavior is key [9]; thus, understanding the impact of salt reduction on food preferences and food intake is relevant [10], [11].

In adults, a positive association was found between perceived saltiness and preferences for snack foods with varying salt contents, and a positive link was also found between liking salty snacks and dietary sodium intake [12]. Moreover, saltiness impacted preferences differently depending on the type of food tested, supporting the hypothesis that there are food-specific preferences for high salt content [8], [13]. In addition, another study of adults found a different impact of salt content on preferences and intake: researchers compared the intake of foods with varying salt content to their hedonic ratings after a sip-and-spit tasting. The optimal salinity revealed by the hedonic rating was higher than the optimal level revealed by the intake data [14]. A study of 10- to 12-year-old children showed that those who liked salty foods (as reported by their mother) ingested more salt (as estimated by urinary sodium excretion) [15]. Another study showed a positive effect of salt content on food intake in 2- to 3-year-olds [16]. However, it could not address the role of hedonic responses in children this young for methodological reasons [17]. One might wonder if the food intake of older children would reflect the same response to salt content variations and to what extent their intake would relate to their liking of the food. The objective of the present study is thus to evaluate the impact of salt content on school-age children’s food intake and food hedonic ratings. Based on the results observed with the toddler population [16], one would hypothesize that salt content would also have a positive effect on intake in an older group of children (e.g., children aged 8–11 years) and that this effect would be food specific. To the best of our knowledge, such an investigation of the influence of salt content on food hedonic ratings and intake has never been conducted in the same group of children.

Thus, the first objective of the present study was to evaluate the effect of salt content on school-age children’s measured intake and hedonic responses for two foods (green beans representing vegetables and pasta representing grains). The second objective was to determine the relative importance of several factors potentially influencing children’s food intake (salt content, hedonic responses, hunger and weight status).

## Materials and Methods

This study used a within-subject crossover design. It was conducted from October to December 2009 in Dijon (France).

### 1. Participants

#### Ethics statement

The study was conducted according to the guidelines laid down in the Declaration of Helsinki and was approved by a local ethics committee (Comité de Protection des Personnes Est I Bourgogne, 2009/39; AFSSAPS ID RCB: 2009-A00966-51). The study took place in one school in Dijon (France). Parents received written information about the study and allowed their child to participate with a signed consent form. Two sets of parents refused to allow their child to participate.

Parents provided information about their child’s body weight (kg) and height (m) based on their child’s health records; z-scores for BMI (z-BMI) were then calculated [18]. The sample included a total of 106 children between the ages of 8 and 11 years. Their parents’ socioeconomic status ranged from mid-level to high.

### 2. Experimental Design

On the basis of a previous study conducted with toddlers, in which salt content modification had a significant impact on green bean and pasta intake [16], a power analysis [19] showed that 33 children were necessary to detect a difference of 8 g of green bean intake between an unsalted variant and a variant with 0.6% added salt (setting the significance level at 0.05 for type I error and the power at 0.80).

The children’s intake was monitored five times in a school cafeteria at lunchtime. After the five lunches, the children came to the laboratory to evaluate the two target foods. The children were blinded to the aim of the study. The parents were asked not to share the study aims with their children. The school staff members were aware that salt content was modified but were blinded to the experimental design.

#### Test foods

The target foods in which the salt content was manipulated were green beans and pasta. These foods are usually served in the school cafeteria with added salt and butter. The three salt conditions were the same as those used in a previous study with 2- to 3-year-old toddlers [16]: no added salt, 0%; 0.6 g of salt added to 100 g of food; or twice this quantity, 1.2 g of salt added to 100 g of food. These levels correspond to 0, 240 and 480 mg of sodium per 100 g of green beans and to 5, 245 and 485 mg of sodium per 100 g of pasta, respectively. The intermediate salt content (0.6%) is the same level that is typically used for foods in school cafeterias and was thus considered the reference salt level. Unseasoned frozen green beans (CMI Carrefour, Les Ulis, France) and dried pasta (Fusilli, Panzani, Lyon, France) were boiled with tap water, without adding salt, oil or any flavoring. Unsalted butter (2.5 g per 100 g of food) was added to the food for all conditions. Salt was added by the experimenters according to the experimental design. The food and ingredients were stirred thoroughly to ensure a homogenous mixture.

#### Experimental lunches: measurements of food intake

The intake measurements were taken every other week at lunchtime (between 11:45 am and 12:45 pm). Salt shakers and salted seasonings (ketchup, mustard) were not available during the experiment. Intake of each food on the menu (serving minus leftovers, in g) was measured for each child to the nearest 1 g (Soehnle, Leifheit Group, Germany). The first observation involved the control condition, in which the green beans and pasta were prepared with the intermediate salt content. For each of the following observations, the salt content varied only in the green beans or in the pasta. For instance, if the salt content was different from 0.6% in the green beans, the pasta was served with 0.6% salt content.

An experimental fixed menu was set. All the menu items were chosen because they were regularly consumed at the school cafeteria and at home. They included a vegetable salad (a single serving of 50 g; 0.87 kcal/g or 3.64 kJ/g; 3.7 mg of Na/g), chicken (a single serving of 50 g; 2.26 kcal/g or 9.46 kJ/g; 4.2 mg of Na/g), green beans (an initial serving of 100 g and a second serving of 100 g; 0.37 kcal/g or 1.55 kJ/g), pasta (an initial serving of 100 g and a second serving of 100 g; 1.4 kcal/g or 5.86 kJ/g), cheese (a single serving of 18 g; 2.39 kcal/g or 10 kJ/g; 7.8 mg of Na/g; La vache qui rit®, Fromageries BEL, Paris, France), fruit puree (a single serving of 100 g; 0.57 kcal/g or 2.38 kJ/g; 0.1 mg of Na/g; Hero®, Allex, France), bread (*ad libitum*; 2.65 kcal/g or 11.1 kJ/g; 6.5 mg of Na/g) and water (*ad libitum*). The portion sizes were adapted from the French guidelines for school canteen usage [20]. The vegetable salad (or macédoine) was a mixture of vegetables, including peas and small pieces of green beans, carrots and turnips; it was prepared with mayonnaise (7 g/100 g). The chicken was poached and grilled in the oven. Both foods were prepared by the canteen cook as usual, following the same recipe for all five lunches. The foods were pre-portioned by the experimenters before lunchtime and kept warm (+63°C) until lunchtime. The children were free to ask for one additional serving of both target foods, as was customary at the school, and the experimenter documented any additional servings. The leftovers from each child were measured in a kitchen area out of the children’s sight, so that they were not aware that their food intake was monitored.

#### Sensory evaluation

The children participated in a sensory evaluation session to evaluate the three variants of each target food. They gave a hedonic rating, a preference ranking and a perceived saltiness ranking. This sensory evaluation session was organized as part of a school outing. It took place at 10:00 am at the Centre des Sciences du Goût et de l’Alimentation laboratory because this is the time of day when children are most alert to perform sensory testing [21]. During this session, the group was divided into two sub-groups: one group started with pasta samples, and the other group started with green bean samples to counteract potential order effects. First, each sub-group gave their hedonic rating of the food on a five point facial scale (from ‘very bad’, −2, to ‘very good’, +2) [17]. The samples were presented one at a time, following a Williams Latin Square design. The children were instructed to rinse their mouths with water between the two samples (Evian® water, France; 6.5 mg of sodium/L). Second, they were served the three variants consecutively and asked to rank them according to their preference (from ‘most preferred’, +1, to ‘least preferred’, −1) and then according to their saltiness (from ‘most salty’, +1, to ‘least salty’, −1). Ties were not allowed. In this study, both an overall hedonic rating and a preference ranking were measured because these two variables provided us with different information about the child’s hedonic responses. The hedonic rating score made it possible for a child to score a food poorly, regardless of its salt content, if s/he did not like it in general. The preference ranking ‘forced’ the child to decide which variant was preferred, regardless of her/his opinion of the given food. The foods were kept warm after the salt was added, and 30 g was served in a 6 oz. (175 ml) isotherm-covered cup (45°C).

### 3. Statistical Analysis

First, univariate analyses were carried out to investigate the effects of salt content and z-BMI on target food intake, hedonic score and preference rankings. Second, a multivariate analysis was performed using Structural Equation Modeling (SEM) to evaluate the respective weights of the different variables in influencing the intake of the target foods. The significance level was set at *p*<0.05.

#### Univariate analyses

The analyses were carried out using SAS Software (9.1, SAS Institute Inc., Cary, NC, USA). Intake (g) and hedonic score data for both target foods were analyzed using a linear mixed model (SAS Mixed procedure), with ‘child’ considered as a random effect. The primary factor tested in the model was salt content, and z-BMI was introduced as a covariate. The model was the following: Intake (or hedonic rating) = food+salt content+z-BMI+food × salt content+food × z-BMI+salt content × z-BMI+error. The ‘Empirical’ option was specified to include a sandwich estimator for the variance-covariance matrix of the fixed-effect parameters. If a significant food × salt content interaction was found, the data were analyzed for each target food separately using the mixed linear model: Intake (or hedonic rating) = salt content+z-BMI+salt content × z-BMI+error. The preference rankings and saltiness rankings for each food were analyzed according to the model: Ranking = salt content+error.

If a significant effect was revealed, multiple mean comparisons were applied using the Dunnett test: the reference salt content (0.6% added salt) was compared to the other variants. The data are reported as means ± SEM.

#### Structural equation modeling (SEM) analysis

Structural Equation Modeling and bootstrap analyses were conducted with R2.10.1, using the SEM package [22] to assess a causal pathway derived from theory [23].

#### Dataset structure and variable definition

The SEM method can handle multilevel data [24]. In this study, the following variables were taken into account: intake (of vegetable salad, chicken, green beans and pasta), hedonic score and preference ranking (of green beans and pasta with different salt contents), salt content and z-BMI. There were two levels of data, as some variables were recorded at the meal level (level 1; intake of each food item), whereas others were recorded at the child level (level 2; hedonic rating or preference ranking for green beans or pasta with a given salt content, z-BMI). For green beans and pasta, the hedonic rating of the variant with 0.6% added salt was used as a measure of the ‘overall hedonic rating’ of each food, and the hedonic rating difference relative to the variant with 0.6% added salt was used as a measure of ‘relative rating’. A latent construct called ‘preference’ was defined using the observed variables of preference ranking and relative assessment, as they actually measure the same construct. One latent construct (‘preference’) was defined for each target food. Another latent construct called ‘hunger’ was defined at the meal level and was thought to provide information about the variability in intake from a child to another. This construct was thought to have an impact on the child’s intake of both target foods and on the intake of all foods consumed before them or at the same time (vegetable salad and chicken).

Because we took into account the variability in children’s intake using the latent construct ‘hunger’, and given the recognized relationship between food intake and BMI [25], we hypothesized that z-BMI would be related to the ‘hunger’ construct.

Thus, the modeling approach, based on a correlation matrix, allowed us to evaluate and compare the variables influencing food intake [23]: salt content, hedonic rating and preference ranking, the child’s ‘hunger’ and z-BMI. Pearson (when both variables were continuous), polyserial (when one variable was discrete and the other was continuous) or polychoric correlations (when both variables were discrete) were calculated [26].

#### Model construction and estimation

The maximum likelihood estimator was used to estimate the model parameters (loadings). The first model, based on theoretical considerations, included an effect of the overall hedonic rating of green beans and pasta (i.e., a rating of the 0.6% added salt variant) on intake; this effect occurred at the child level. It also included the latent construct ‘preference’. The effect of ‘preference’ on intake was considered for both target foods. A quadratic effect of salt content on ‘preference’ was added for green beans and pasta. Thus, the salt content variable and its square were included after orthogonalization [27]. Thereby, we considered that a higher salt content could be related to a higher preference ranking, which in turn could be related to higher intake.

The same scheme was used for green beans and for pasta. The correlation between the overall and relative hedonic ratings was included. The correlation between the overall hedonic rating of green beans and the overall hedonic ratings of pasta was added to the model to account for inter-rater differences in using the hedonic ratings scale. The effects of vegetable salad intake on the intake of green beans, pasta and chicken were added (set to the same value) to account for the fact that a child who had eaten a large amount of salad might not have been as hungry for the remainder of the meal.

The nested model obtained by applying equality constraints to the green bean and pasta parameters (the effect of salt content on ‘preference’, the effect of ‘preference’ on intake, the effect of the overall hedonic rating on intake, ‘preference’ loadings on preference ranking and relative hedonic rating) was compared to the unconstrained model.

Finally, the effect of z-BMI on ‘hunger’ was investigated, hypothesizing that higher z-BMI would correlate with higher overall intake. Next, modification indices were considered to improve the model, and they led us to consider a specific z-BMI effect on the hedonic rating and intake of pasta. When these two effects were introduced into the model, the z-BMI effect on ‘hunger’ was no longer significant, suggesting a specific effect of z-BMI on the hedonic rating and intake of pasta rather than a general effect on ‘hunger’. Because this result was consistent with a previous observation in toddlers [16], this modification was implemented in the model. The modification indices also suggested a link between the overall hedonic rating of green beans and vegetable salad intake; this link was added to the model. This effect makes sense because the vegetable salad included pieces of green beans.

Comparisons between the theoretical model and the modified models were tested with likelihood ratio tests (p<0.05).

Because the observations were clustered by child, the assumption of independent observations was not met. Therefore, the standard errors and 95% confidence intervals of the parameters were obtained using bootstrap resampling (B = 100), with the resampling performed at the child level [24], [28].

#### Model validation and indicators of model fit

The fit is considered good when the model is parsimonious and when the fitted covariance matrix is close enough to the observed covariance matrix. As recommended, several fit indices that address different aspects of model fit are reported here: the chi-square test, the Tucker-Lewis Non-Normed Fit Index (NNFI), the Bentler Comparative Fit Index (CFI) and the Root Mean Square Error of Approximation (RMSEA). The RMSEA is expected to be as close as possible to 0, with a value of about 0.05 considered good, while the NNFI and the CFI are expected to be as close as possible to 1, with values of about 0.90 considered acceptable. The chi-square statistic is also reported as a relevant index, but it was not used to perform a formal test because models are usually rejected [29].

## Results

Data for 75 children (35 girls) were taken into account in the present analysis. Three hundred forty-nine meals were recorded. One, 3, 17 and 54 children took part in 2, 3, 4 and 5 meals, respectively. The children’s mean age was 9.4±0.1 years (range: 8 to 11 years), with a mean BMI of 17.1±0.3 kg/m^2^ and a mean z-BMI of 0.49±0.15 SD (range: 2.28 to 4.23 SD). Of these children, 11% were considered overweight or obese (z-BMI>+2 SDs) [30].

Over the five lunches, the average total intake was 367±9 g (range: 137 to 520 g), and the average total energy intake was 412±9 kcal (range: 172 to 570 kcal) or 1723±38 kJ (range: 720 to 2384 kJ). A paired *t* test showed that over the five lunches, green bean intake (75±4 g; range: 4 to 160 g) was lower than pasta intake (136±5 g; range: 61 to 200 g) (t *_74_* = 10.48; p<0.0001).

### 

#### Univariate analysis: The impact of salt content on intake

**Figure 1:**
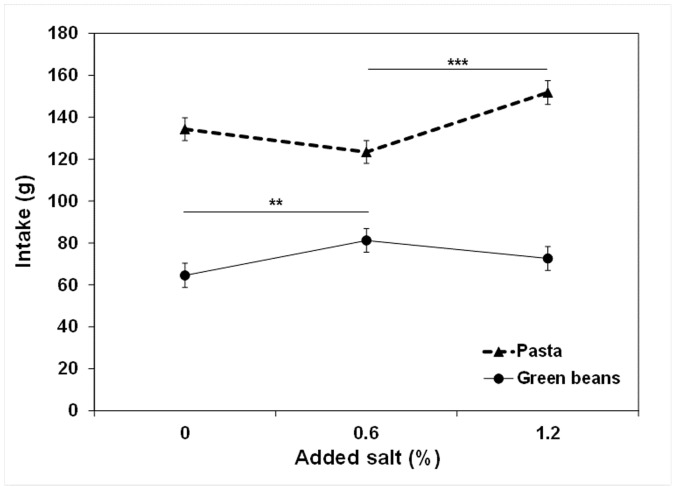
The impact of added salt on green bean and pasta intake by 8- to 11-year-olds. The variations tested were 0, 0.6 or 1.2 g of added salt per 100 g of food. The data are presented as least squares means with SEM. The 0% and 1.2% added salt variants were compared to the 0.6% level with a Dunnett test: ** p<0.01 and *** p<0.001.

A significant food x salt content interaction effect on food intake was found (F (2, 271) = 15.24; p<0.0001). Further analyses were run separately for each target food. The salt content impacted green bean and pasta intake significantly (F (2, 137) = 5.65; p = 0.0045 and F (2, 134) = 12.26; p<0.0001, respectively) and differently, as fewer unsalted green beans were consumed than green beans with 0.6% added salt, whereas more of the pasta with 1.2% added salt was consumed than the pasta with 0.6% added salt (**Figure 1**). In the case of green beans, the significant difference between the intake of the 0.6% variant and the intake of the 0% variant results in a 200 mg difference in sodium intake or a 0.5 g difference in salt intake. In the case of pasta, the significant difference in between the intake of the 1.2% variant and the intake of the 0.6% variant results in a 280 mg difference in sodium intake or a 0.7 g difference in salt intake.

Green bean intake was not related to z-BMI, whereas pasta intake was higher when z-BMI was higher (F (1, 137) = 0.08; p = 0.77 and F (1, 134) = 18.21; p<0.0001, respectively). For a 1 SD increase in z-BMI, pasta intake increased by 15 g. The effect of the salt content x z-BMI interaction on intake was neither significant for green beans (F (2, 137) = 0.01; p = 0.99), nor for pasta (F (1, 132) = 1.07; p = 0.35).

#### Univariate analysis: The impact of salt content on hedonic rating and perceived saltiness

**Figure 2:**
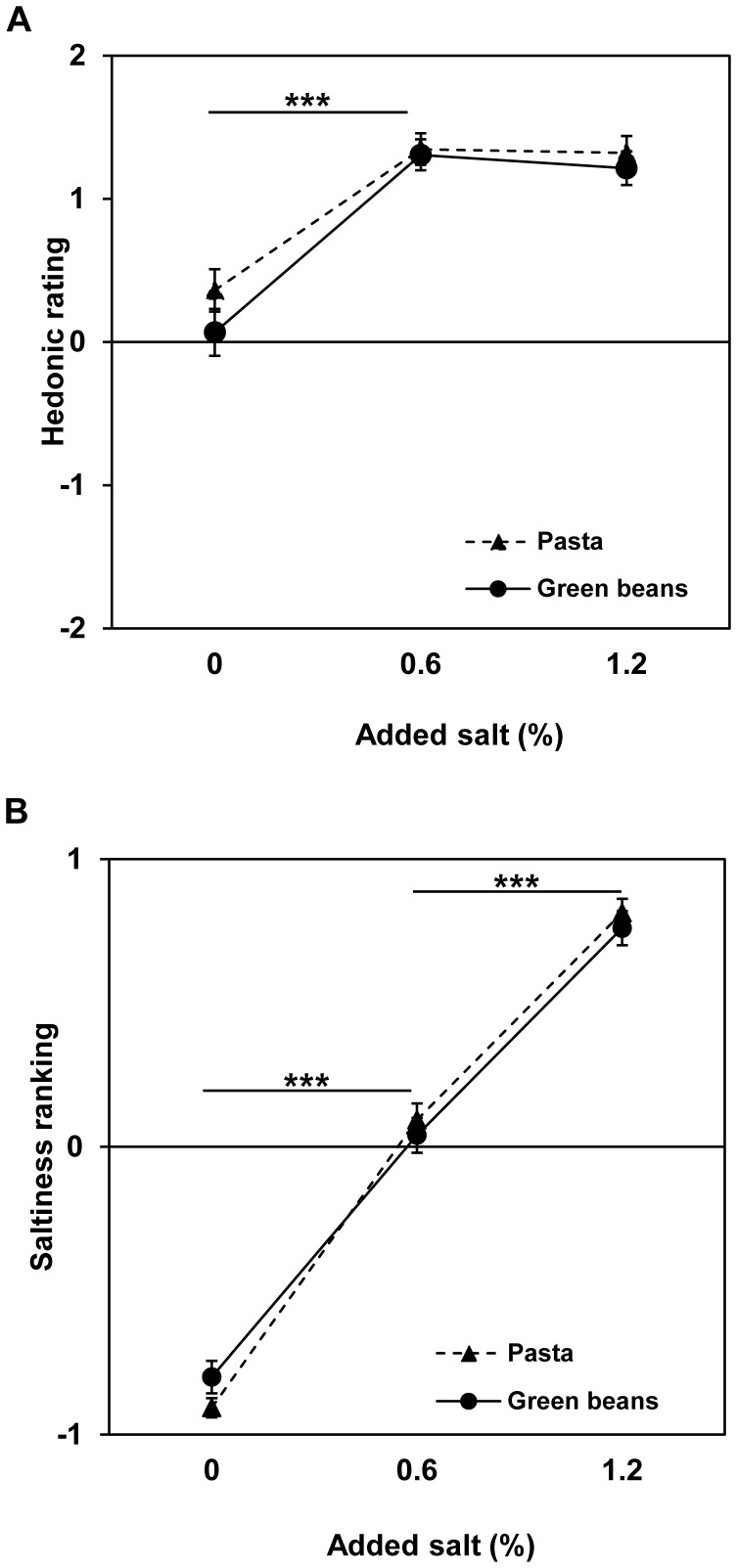
The impact of added salt on green bean and pasta sensory evaluation by 8- to 11-year-olds. The variations tested were 0, 0.6 or 1.2 g of added salt per 100 g of food. Least squares means with SEM are presented for (A) hedonic rating scores (from ‘very bad’, −2, to ‘very good’, 2) and (B) saltiness rankings (from ‘least salted’, −1, to ‘more salted’, 1). The 0 and 1.2% added salt variants were compared to the 0.6% level with a Dunnett test: ** p<0.01 and *** p<0.001.

For the hedonic rating data, the food effect (F (1, 74) = 2.79; p = 0.10) and the interaction between food and salt content (F (2, 296) = 1.17; p = 0.31) were not significant. The food factor was removed from the model, and only the salt content factor was taken into account. The variants with 0.6% and 1.2% added salt were liked more than the unsalted variant (F (2, 298) = 32.32; p<0.0001; **Figure 2A**). In the initial model, the effect of the interaction between food and z-BMI on the hedonic rating of the food was significant (F (1, 292) = 4.52; p = 0.035), but in separate models for each food, the effect of z-BMI on the hedonic rating was not significant (F (1, 136) = 1.22; p = 0.27 for green beans and F (1, 156) = 0.27; p = 0.60 for pasta). The effect of the salt content x z-BMI interaction on hedonic rating was neither significant for green beans (F (2, 136) = 0.87; p = 0.42), nor for pasta (F (2, 156) = 0.66; p = 0.52).

For the ranking data, the salt content factor was significant. For both target foods, the variants with 0.6% and 1.2% added salt were preferred over the unsalted variant (F (2, 298) = 53.48; p<0.0001). The mean rankings (with scores varying from −1 to 1) for the variants with 0%, 0.6% and 1.2% added salt were −0.61, 0.39 and 0.23 for the green beans and −0.60, 0.24 and 0.36 for the pasta, respectively. When asked to rank their perceptions of saltiness, the children were able to distinguish the three variants for both foods (F (2, 298) = 328.86; p<0.0001; **Figure 2B**).

#### Structural equation modeling: The relationship between salt content, hedonic rating and intake

**Figure 3:**
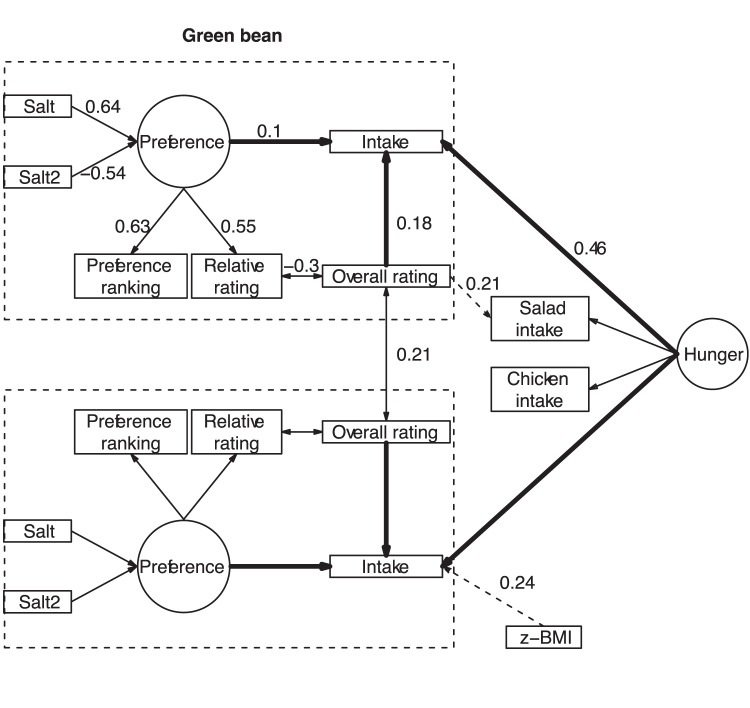
Structural Equation Model illustrating the drivers of children’s green bean and pasta intake. The SEM assessed the relationships between salt content, green bean and pasta preferences and intake, hunger and z-BMI. SALT2 = Salt^2^. Salad is vegetable salad. The variables in squares represent measured/observed variables, and those in circles represent latent variables. The effects concerning green beans are set equal to those concerning pasta and are reported for green beans only. The effect of hunger on the intake of green beans, pasta, salad and chicken were set to the same value (reported only once). Dotted lines represent effects suggested by the modification indices, and solid lines represent assumed effects. Numerical values next to arrows represent path coefficients, significant at p<0.05. All fit indices suggest a good fit: model chi-square = 135, df = 85, p = 0.0005, Tucker-Lewis NNFI = 0.93, Bentler CFI = 0.95, RMSEA = 0.04 with 90% confidence interval [0.03, 0.06].

SEM was used to test the theory that salt content influences intake through its impact on preferences. The final model is represented in **Figure 3**. All of the fit indices suggested a good fit. The model explained 21% and 26% of the variance in green bean intake and pasta intake, respectively. The results were the same without the equality constraints placed on the green bean and pasta parameters. Bootstrap resampling showed that all of the parameters were significant (p<0.05) except the effect of z-BMI on the overall pasta hedonic rating (p = 0.10), which was suggested by the modification indices.

The primary determinant of intake was the latent construct ‘hunger’ (r = 0.46), followed by the overall hedonic rating of the food (r = 0.18) and the latent construct ‘preference’ (r = 0.10), which was related to salt content for both green beans and pasta. z-BMI impacted pasta intake: the higher the z-BMI, the higher the pasta intake.

## Discussion

The present study aimed to evaluate the impact of salt on children’s food intake, taking into accounts the roles of hedonic responses, hunger and weight status. It showed a significant positive effect of salt content on the intake of green beans and pasta, although the impact was different for these two foods. A reduction in the salt content of green beans decreased their intake by 21%, whereas an increase in the salt content in pasta increased its intake by 24%. Even though the children were able to distinguish between three variants of foods according to their saltiness, they preferred those with more added salt over the unsalted versions of both foods. As expected, the results of the SEM showed that salt modulated food intake by modifying preferences and allowed us to evaluate the weight of the different factors that affected target food intake: 1) the child’s hunger, 2) the child’s hedonic rating of the food, regardless of its salt content and 3) the child’s rating about the particular salt content of the food.

In this study, the effect of salt content on sensory perceptions (hedonic rating, preference ranking and perceived saltiness) and on intake was investigated in real world conditions. To date, few studies have compared hedonic evaluation and intake data by varying the sensory attributes of a single food and using the same subjects. Using adult participants, Bellisle and Lucas compared sensory evaluation data and intake of yogurt that varied in sugar content and of mashed potatoes that varied in salt content [14], [31]. These experiments showed that the optimal amounts of these ingredients (sugar and salt) in a given food are overestimated if the results from the sensory evaluation are the only ones considered. We drew the same conclusion for children and pasta. The minimum salt content needed to optimize hedonic rating scores and preference rankings would be 0.6% added salt in both foods. Based on intake, it seems possible to eliminate salt from pasta because the variant with 0% added salt was consumed as much as the variant with 0.6% added salt and in a large quantity (134 g). For green beans, further research may be needed to explore the impact of a reduction in salt to a level below 0.6%. It may be possible to reveal an intermediate salt content level between 0% and 0.6% that would not negatively impact intake.

Previous studies showed that the perception and acceptability of saltiness might be product-specific [8], [12], [13], [32] and that the preference for a salty taste cannot be generalized to all foods [13], [33]. The present results from intake data are in accordance with these previous findings. The differences in the impact of the salt content of various foods could be due to a different impacts of salt content on the sensory characteristics associated with the food [34]. For the foods in this study, measures of taste intensity obtained with a trained panel reported elsewhere [16] revealed that the salt content affected the intensity of saltiness similarly in both foods but affected other sensory characteristics in the green beans only. Unsalted green beans were perceived as more sour and less fatty than the two other variants. These differences may explain why the absence of salt in green beans resulted in a lower intake of green beans. One may also suppose that impairments of the sensory characteristics are more detrimental for foods that are not consumed in large quantities than for foods that are consumed in large quantities. Altogether, these results suggest possibilities for nutritional improvements related to the salt content in foods targeted at children, particularly salty foods that are easily accepted (e.g., chips, crackers). Taking into account the recommendations and the current sodium intake of the child population [6], [7], a decrease of one gram of dietary salt intake is needed. The present study shows that switching from the 0.6% to the 0% added salt variants of green beans and pasta decreases salt intake by 0.49 g (196 mg of sodium) and 0.72 g (288 mg of sodium), respectively. This change represents a significant decrease in sodium (−484 mg) and a reduction in salt of 1.21 g, which align with nutritional objectives for the child population.

In other respects, the Structural Equation Modeling method was used to better understand the effect of salt content on intake by providing evidence that the relationship is mediated by preferences. This method revealed that a child’s intake of a food is less influenced by liking a specific salt content than by the overall hedonic rating of the food, regardless of its salt content. Using a model integrating intake data from the whole meal (and not only from the target foods) made it possible to capture the ‘hunger’ level. The ‘hunger’ construct was highly varied among the children, as was the individual mean intake over the five meals, which ranged from 137 to 520 g. This result confirms former studies that highlighted the great variability of food intake among children [35]. In this study, the SEM explained 21% and 26% of the variance in the intake of green beans and pasta, respectively. Using the SEM method, Hayes and colleagues [12] found that the hedonic rating of a series of salted chicken broths explained 18% of the variance in the intake of high sodium foods (as measured with a food frequency survey), which is in the same range as the values obtained in the present experiment. Our model could have been improved by including variables that were difficult to measure in the context of the present work, such as parental guidance regarding food consumption or children’s cognitive attitudes towards the target foods. Nevertheless, this modeling approach improves our understanding of the positive but moderate role that salt content plays among the other factors that influence intake.

We showed that z-BMI was specifically related to pasta intake and not to ‘hunger’, as we considered in the initial model. This result should be confirmed with a new and independent dataset to reach a definitive conclusion. Nevertheless, this effect is in accordance with a previous observation that heavier 2- to 3-year-old children tend to consume more pasta [16]. It is possible that heavier children seek energy-dense foods: greater BMI correlates with greater intake required to satisfy energy needs [25].

The results of the present study should be interpreted in light of its strengths and limitations. The study used familiar foods and took place in the children’s usual eating context with their classmates and caregivers. This context remained constant over the study duration. Moreover, the intake and hedonic rating data were collected from the same group of children. In other respects, this work has also some limitations. First, children’s intakes may have been constrained by the fixed menu; the children could have chosen to eat other saltier foods if they had been available. Another limitation is related to the fact that it is difficult to generalize the present findings to other foods (other vegetables, other starchy foods or other salty foods) because the salt content effect appears to be food specific. To further understand the role of salt content, future research should study the impact of low salt content (i.e., between 0% and 0.6%) on the intake of green beans or other vegetables to maximize intake while maintaining palatability. Future studies could also focus on studying the impact of reducing salt content in processed foods that are rich in sodium. The findings of the present study were obtained with a group of children of middle to high SES and may reflect specific habits of salt use. Therefore, it is difficult to generalize the results to children from other social backgrounds. However, the present findings are consistent with results obtained from younger French children representing a wider range of SES [16]. In addition, one may wonder whether adding butter (2.5%) to the target foods may have modified the intake. However, previous results showed no impact of adding butter on the intake of green beans or pasta by 2- to 3-year-olds [16], and the presence of butter was unlikely to have significantly impacted the present findings.

In conclusion, this study with school-age children showed that compared to the usual salt content, adding no salt decreased green bean intake, and increasing the salt content increased pasta intake. It also revealed that considering preferences to determine the optimal salt content could increase salt content beyond what seems necessary for the food to be consumed. This appears to be particularly true for foods that are commonly consumed by children, such as pasta. Thus, food product optimization should consider the importance of real intake data and relativize the importance of hedonic response data.

The present study suggests that in the current context, where many of the foods eaten by children are industrially processed and contain added salt, there may be possibilities for nutritional improvements by reducing salt content. Toward this aim, the present results suggest that it may be relevant to adapt salt reduction to the specific type of food. Salt content could easily be reduced below the current content in palatable foods, whereas moderate salt content is sufficient but necessary in less palatable foods, such as vegetables. To promote such an effort by the food industry, national regulations could be relevant. Moreover, a regulation to reduce added salt in all processed foods could be useful to avoid possible shifts toward salty options. This is one approach that has been implemented in France through charters of commitment of voluntary nutritional improvement with the food industry [36].
